# Body Weight and ADHD: Examining the Role of Self-Regulation

**DOI:** 10.1371/journal.pone.0055351

**Published:** 2013-01-29

**Authors:** Zia Choudhry, Sarojini M. Sengupta, Natalie Grizenko, William J. Harvey, Marie-Ève Fortier, Norbert Schmitz, Ridha Joober

**Affiliations:** 1 Department of Human Genetics, McGill University, Montreal, Canada; 2 Department of Psychiatry, McGill University, Montreal, Canada; 3 Department of Kinesiology and Physical Education, McGill University, Montreal, Canada; 4 Douglas Mental Health University Institute, Montreal, Canada; The University of Queensland, Australia

## Abstract

**Objective:**

Attention-Deficit/Hyperactivity Disorder (ADHD) is a complex and heterogeneous childhood disorder that often coexists with other psychiatric and somatic disorders. Recently, a link between ADHD and body weight dysregulation has been reported and often interpreted as impaired self-regulation that is shared between the two conditions. The objective of this study is to investigate the relation between body weight/BMI and cognitive, emotional and motor characteristics in children with ADHD.

**Methods:**

284 ADHD children were stratified by weight status/BMI according to WHO classification and compared with regard to their neurocognitive characteristics, motivational style, and motor profile as assessed by a comprehensive battery of tests. All comparisons were adjusted for demographic characteristics of relevance including, socioeconomic status (SES).

**Results:**

Both Obese and overweight ADHD children exhibited significantly lower SES compared to normal weight ADHD children. No significant differences were observed between the three groups with regards to their neurocognitive, emotional and motor profile.

**Conclusions:**

Our findings provide evidence that differences in weight/BMI are not accounted for by cognitive, motivational and motor profiles. Socio-economic characteristics are strongly associated with overweight and obesity in ADHD children and may inform strategies aimed at promoting healthier weight.

## Introduction

Attention-Deficit/Hyperactivity Disorder (ADHD) is an etiologically complex, highly heritable, common childhood psychiatric disorder with approximately 5% prevalence worldwide [1]. It is characterized by age inappropriate patterns of severe and persistent inattention, hyperactivity, and impulsivity. ADHD often coexists with other psychiatric [2] and somatic disorders [3].

Recently, ADHD has been associated with body weight dysregulation [4], [5]. Indeed, it has been reported that ADHD subjects show higher than average body mass index standard deviation scores, and have significantly higher percentage of body fat and abdominal circumference compared to controls [6], [7]. Conversely, obese subjects are more likely to present with attention problems [8]. In addition, some evidence suggests that patients with eating disorders tend to have attention problems akin to ADHD [9], [10].

From a psychological point of view, both ADHD and childhood obesity have been conceptualized as disorders of impaired self-regulation [11], [12]. Self-regulation is a psychological construct encapsulating the means by which individuals manage themselves in order to attain adaptative goals [13]. This construct implicates emotional and cognitive, particularly executive function (EF) processes [14], [15]. EF represent the neurocognitive processes important for goal-directed behaviours [16] including planning, sustained attention, cognitive flexibility, working memory, and response inhibition. Deficits in EF are believed to play an important role in ADHD. More specifically, ADHD children have shown significant impairments in different EF domains including response inhibition, vigilance, working memory, and planning [17]. Likewise, extremely obese individuals demonstrate significant differences in performances on tests of executive functioning such as planning, problem solving, and mental flexibility [18]. Similarly, in comparison to their peers, obese children exhibit significant cognitive deficits in their attention shifting abilities [19]. Furthermore, in some studies, adults with elevated BMI display reduced cognitive performance [20]. However, in a recent large longitudinal study, obesity indices were differentially associated with performance on neuropsychological tests; while global cognitive function, memory and language ability were found to be associated with obesity indices, attention and visuospatial ability showed the reverse trends [21].

In addition to EFs, self-regulation depends critically on motivational systems that may be dysregulated in both ADHD and obesity. In this regard, mesolimbic dopaminergic neurotransmission has been firmly implicated in the regulation of saliency of behavioral tasks [22] and food stimuli [23]. It has been proposed that ADHD symptoms may be at least in part the consequence of reduced dopamine (DA) signaling in the mesolimbic system, resulting in reduced saliency of tasks [24] and aversion to delayed gratification [25]. This emotional style may result in preferring immediate rewards and being less sensitive to reinforcement schedules [26], [27], and consequently may contribute to abnormal eating habits [28] and promote inclination towards readily available non-healthy food in patients with ADHD. Similarly, pathological eating behavior (food addiction) in obese individuals has been conceptualized as a reaction compensating for a dampened DA reward system [29], [30]. Under this scenario, excessive food intake may result in elevated dopamine activation in the mesolimbic pathway leading to a rewarding experience [31], [32].

These neurocognitive/emotional parallels between ADHD and obesity are also supported by neuroimaging studies in ADHD and obese subjects [33], [34], [35], [36]. Further, it is also noticeable that psychostimulant medications, used to treat ADHD symptoms, also reduce appetite [37], [38]. These observations taken together suggest that the neurobiological pathways implicated in the regulation of attention and motor control are also important determinants of energy intake and eating behaviors [39], [40]. While the literature linking ADHD to body weight regulation is rich in neuropsychological hypotheses attempting to explain the link between these two complex disorders, the experimental data supporting these theories is very limited. To our knowledge, only one study reported an association between body weight and EF in children with ADHD [12]. In addition, the association between ADHD and body mass dysregulation could be due to many factors shared by these two conditions. Childhood socioeconomic adversity has been shown to impact both ADHD [41], [42] and obesity [43]. More specifically, in a national cohort study with a sample of 7,960 children (aged 6–19 years), familial socioeconomic adversity factors including low maternal education level, single parent status and being recipient of social welfare predicted medicated status in school children with ADHD [42]. Likewise, in developed countries, groups belonging to low socioeconomic status (SES) are more likely to be obese compared to other SES groups [44], [45], [46].

The present study aims at testing the hypothesis that self-regulation deficits are associated with obesity in children with ADHD. Further, our study controls for socioeconomic status, consists of a large sample size, and utilizes a comprehensive assessment battery.

## Methods

### Ethics Statement

The research protocol was approved by the Research Ethics Board of the Douglas Mental Health University Institute (DMHUI). Children with ADHD and their parents were explained the study procedures in detail, and provided verbal assent and written consent respectively.

### Subjects and study procedure

Two hundred and eighty four children with ADHD (210 males and 74 females), ages 6–12 [mean = 9.15; SD = 1.86], were recruited from the severe disruptive behaviours program and ADHD outpatient clinic at the DMHUI. These children were referred to the aforementioned secondary care mental health facilities from different sources including, school teachers, pediatricians, family physicians, and community social workers. Thus the children participating in the current study are reflective of the general ADHD population.

All children included in this study met DSM-IV diagnosis criteria for ADHD. A comprehensive clinical evaluation was used to establish the diagnosis of ADHD [see details in [47]. Children were excluded from this study if they had an IQ less than 70 on the Wechsler Intelligence Scale for Children-III/IV (WISC-III or WISC-IV), Tourette syndrome, pervasive developmental disorder, or psychosis. These subjects were excluded from the study in-order to reduce the potential confounding of cognitive and weight by the afore-mentioned medical conditions, which have been associated with weight gain [48], [49], [50] and cognitive anomalies [51], [52], [53], [54].

Among the total sample of affected ADHD children, 73.9% were male, and 18.0% belonged to families with an annual income of less than CN$20,000, 49.3% met *DSM-IV* criteria for the combined subtype, 42.3% were diagnosed with the inattentive and 8.1% with the hyperactive subtypes of ADHD. A total of 30.3% were previously receiving medication for their ADHD symptoms and the rest were medication naive. Among comorbid disorders, 41.9% had oppositional defiant disorder, 11.3% had conduct disorder (CD), and 40.1% had an anxiety disorder.

All the clinical and self-regulation assessments were completed while the children were not taking any medication. In cases where children were on medication prior to their inclusion in the study, these assessments were carried out at the end of a one-week washout period.

### Self-regulation evaluations

#### Neurocognitive performance

A comprehensive neuropsychological (NP) battery of tests specifically designed for children was used to study different executive function domains. Amongst the NP test battery, the Wechsler Intelligence Scale (WISC) [55] evaluates the full scale (FS), verbal (V), and performance (P) IQ; the Wisconsin Card Sorting Test (WCST) [56] assesses cognitive flexibility and set-shifting; the Finger Windows (FW) subtest [57] assesses visual-spatial working memory; the Tower of London (TOL) [58] evaluates planning, organization, and problem-solving capacity; the Self-Ordered Pointing Task (SOPT) estimates working memory, planning, and response inhibition [59]; the Conners' Continuous Performance Test (CPT) [60] measures attention, response inhibition, and impulse control; and the Stroop (colour and word) test evaluates cognitive flexibility, and resistance to interference from outside stimuli [61]. The details regarding NC task assessments and procedures have been described in detail in a previous study [62].

#### Motivational style

Motivational style was first evaluated by using the choice delay task (CDT), a test specifically designed to assess the ADHD children's aversion to delay [63]. In the CDT, the child repetitively (20 trials) chose between two reward paradigms; a large reward of 2 points (exchanged for 7 or 10 cents) associated with a large period of delay (30 sec), and a smaller reward of 1 point (exchanged for 5 cents) associated with a smaller period of delay (2 sec). However, once the participant chose a particular reward paradigm, he/she cannot switch back to the alternative reward paradigm until the next trial [63].

In addition to the CDT, the Restricted Academic Situation Scale (RASS) [64] was used to systematically observe and record the child's engagement in an assigned independent academic task (a set of math problems) in the presence of potential distractions, with no adult supervision [65]. Task engagement/disengagement, is a distinct trait of ADHD [66], [67] and it is also a good predictor of the child's motivation during a monotonous and repetitive task. The RASS assessment was conducted in a specialized room within the clinic equipped with a worktable, a chair, an intercom, and some toys. The child was given a set of math problems at current grade and instructed to complete as many as possible. The instructor then left the room and assessed the child's behaviour from behind a one-way mirror over a 15 minute time period. All behavioural events were recorded at 30-second intervals according to five categories: ‘off-task’, ‘playing with objects’, ‘out of seat’, ‘vocalizing’, and ‘fidgeting’.

#### Motor activity

On the day of the testing, overall motor activity of children with ADHD was evaluated using actigraphy using a small electronic device (Actiwatch®) worn on the non-dominant hand, which is sensitive to acceleration. It records the subject's movements each 30 sec time period and expresses it as motor activity counts. Patients put on the Actiwatch® in the morning and kept it until the end of their testing in the early afternoon. The average motor activity was calculated and considered in the analyses as reflective of the overall motor activity of the child.

### Anthropometrics

All anthropometric measurements were taken by trained research assistants. Height of children with ADHD was measured using a wall-mounted chart with the subjects being bare-footed. Similarly, body weight was measured using a doctor's clinical scale with subjects clad in regular casual attire and being bare-footed.

### Calculation of Body Mass Index (BMI) and definition of weight categories

Body mass index (BMI) is a simple index of weight-for-height used to classify children into normal, overweight and obese categories. It is defined as “weight in kilograms divided by the square of the height in meters (kg/m^2^)”. BMI was calculated for all children with ADHD, and then converted to age- and gender-specific percentiles according to the criterion available in the World Health Organization (WHO) website (http://www.who.int/en/). The WHO defines “normal weight” as ranging between 3^rd^ to 84^th^ percentile, “overweight” as ranging between 85^th^ to 96^th^ percentile, and “obesity” as equal or greater than 97^th^ percentile.

### Statistical Methods

Statistical analyses were performed with SPSS 15.0 version for Windows. Subjects were stratified into three groups according to the Weight/BMI classification as per WHO criterion. First, demographic and baseline characteristics of children with ADHD were compared between the three groups, namely normal (n = 168), overweight (n = 57), and obese (n = 59). Socio-demographic variables associated with BMI at a significance level of p<0.05 were used as covariates in subsequent analyses. Second, neurocognitive, emotional and motor outcome measures were compared between three weight/BMI groups using univariate ANOVA for continuous variables and chi-squared tests for categorical variables. Main effects were further explored by Post Hoc pairwise comparisons and the Bonferroni correction was used to protect from type I error.

## Results

As shown in Table 1, the three groups of children were not different with regard to age, gender, handedness or birth weight. However, mothers and fathers of children in the obese and overweight groups were significantly younger at the birth of their children (F_2,261_ = 5.14, p = 0.006; F_2,233_ = 4.55, p = 0.01, respectively; all pair-wise p-values comparing the normal weight group to the other two groups were ≤0.01). In addition, compared to others, children in the overweight and obese groups belonged to a lower family income group (Χ^2^ = 7.84, df  = 2, p = 0.01), and were also exposed to higher severity of maternal smoking during pregnancy (MSDP; # of cigarettes per day; F_2,248_ = 3.26, p = 0.04).

**Table 1:** Demographic and baseline characteristics of ADHD children stratified according to three BMI categories.

	Normal-weight n = 168	Over-weight n = 57	Obese n = 59	Test statistic, p-value, post hoc comparison	Effect size (Cohen's *d*)	
					Normal- weight vs. Over-weight	Normal- weight vs. Obese
***Subject characteristics***						
Gender (% males)	75.0%	66.7%	78.0%	X^2^ = 2.15, df = 2, p = 0.34		
Age	9.01±1.86	9.08±1.80	9.6±1.86	F_2,281_ = 2.23, p = 0.10	−0.03	−0.31
Handedness (Rt/lft/amb.)	150/18/0	50/7/0	49/8/2	X^2^ = 8.15, df = 4, *p = 0.08*		
Birth weight (gms)	3396.90±711.62	3541±814.951	3269.56±521.03	F_2,126_ = 1.04, p = 0.35	−0.18	0.20
Previous medication status (% yes)	36.1%	25.0%	20.3%	X^2^ = 6.15, df = 2, ***p = 0.04***		
***Family characteristics***						
Participating legal guardian (% mothers)	82.9%	82.1%	91.4%	X^2^ = 2.64, df = 2, p = 0.26		
Mother's age at child's birth	29.51±5.84	28.60±7.10	26.58±4.72	F_2,261_ = 5.14, ***p = 0.006,*** ╪	0.13	0.55
Mother's years of education	13.87±3.28	13.27±3.09	13.51±2.53	F_2,245_ = 0.77, p = 0.46	0.18	0.12
Maternal alcohol during pregnancy (% yes)	24.0%	18.8%	16.7%	X^2^ = 1.52, df = 2, p = 0.46		
Maternal smoking during pregnancy (# of cigarettes per day)	2.43±5.52	4.93±7.73	3.71±6.21	F_2,248_ = 3.26, ***p = 0.04,*** ┼	−0.37	−0.21
Adopted (% yes)	5.4%	3.6%	0.0%	X^2^ = 3.39, df = 2, p = 0.18		
Father's years of education	13.14±3.43	12.41±3.59	12.32±3.51	F_2,202_ = 1.19, p = 0.30	0.20	0.23
Father's age at child's birth	31.95±6.03	30.61±7.17	28.94±5.26	F_2,233_ = 4.55, ***p = 0.01,*** ╪	0.20	0.53
Annual family income (% less than $20,000)	13.4%	29.1%	24.1%	X^2^ = 7.84, df = 2, ***p = 0.01***		

* *Values are mean ± SD unless otherwise specified. Rt  =  right, lft  =  left, amb  =  ambidextrous, gms  =  grams, #  =  number. Significant Post Hoc pairwis; comparison nomenclature;* ┼ * =  normal vs. over-weight, ╪  =  normal vs. obese.*

With regards to previous medication status, ADHD children within the obese group were significantly less likely to be previously on medication (20.3%) compared to subjects in the overweight (25.0%), and normal weight (36.1%) groups (Χ^2^ = 6.15, df  = 2, p = 0.04; Table 1). This finding is in line with previous data indicating that stimulant medication in ADHD subjects may result in weight loss due to suppression of appetite [68], [69].

All subsequent analyses were conducted while controlling for aforementioned SES factors (parental age at child birth, family income status, and MSDP) and prior history of treatment with psychostimulants.

Additionally, as shown in Table 2, neurocognitive features as assessed by the WISC-III, WCST, TOL, SOPT, FW, CPT, and Stroop were not significantly different between the three groups (all p>0.05). Further, no significant differences between the groups were found in emotional/motivational style as evaluated by the CDT and the RASS respectively (all p>0.05; Table 3). Finally, the three groups were not significantly different with regards to motor activity as evaluated by actigraphy (all p>0.05; Table 3).

**Table 2:** Neurocognitive features of ADHD children stratified according to BMI categories.

	Normal-weight n = 168	Over-weight n = 57	Obese n = 59	Test statistic and p-value	Effect size (Cohen's *d*)	
					Normal- weight vs. Over-weight	Normal- weight vs. Obese
**Neurocognitive evaluation**						
***WISC-III***						
verbal IQ	96.53±13.53	97.37±14.37	94.63±14.61	F_2,185_ = 1.27, p = 0.28	−0.06	0.13
performance IQ	103.74±13.79	101.16±14.61	99.45±15.97	F_2,185_ = 0.83, p = 0.43	0.18	0.28
full-scale IQ	96.81±12.27	96.82±13.47	93.82±12.45	F_2,185_ = 0.94, p = 0.39		0.24
***Wisconsin Card Sorting Test***						
Perseverative errors (s score)	97.58±13.23	99.37±15.24	96.91±10.90	F_2190_ = 0.36, p = 0.69	−0.00	0.05
Non-Perseverative errors (s score)	92.25±15.90	96.94±17.35	95.63±15.04	F_2,190_ = 1.56, p = 0.21	−0.28	−0.21
Total errors (s score)	94.31±14.50	96.86±15.00	96.12±12.98	F_2190_ = 0.47, p = 0.62	−0.17	−0.13
number of categories completed	4.14±1.92	4.40±2.06	4.30±1.79	F_2,190_ = 0.31, p = 0.73	−0.13	−0.08
Failure to maintain set	1.48±1.29	1.66±1.51	1.86±2.01	F_2,190_ = 1.15, p = 0.31	−0.12	−0.22
***WRAML Finger Windows***						
Standard score	9.74±2.89	10.23±3.25	9.68±2.63	F_2,204_ = 0.90, p = 0.40	−0.15	0.02
***Tower of London***						
Standard Score	110.39±15.49	112.11±13.22	107.61±18.40	F_2,182_ = 0.97, p = 0.37	−0.11	0.16
***Self-Ordered Pointing Test***						
Total score	16.98±7.61	16.33±7.13	15.77±7.06	F_2,204_ = 0.66, p = 0.51	0.08	0.16
***Stroop Test***						
Word score (t score)	51.37±9.15	51.69±9.64	49.83±7.36	F_2,172_ = 0.36, p = 0.69	−0.03	0.18
Color score (t score)	48.79±6.62	48.41±7.37	47.75±5.42	F_2,172_ = 0.20, p = 0.81	0.05	0.17
Color-Word Score (t-Score)	44.73±8.60	44.78±7.60	44.15±8.01	F_2,172_ = 0.12, p = 0.88	−0.006	0.06
Interference score (t score)	52.56±8.50	52.13±7.10	51.60±7.49	F_2,172_ = 0.06, p = 0.93	0.05	0.11
***Continuous*** ***Performance Test***						0.06
Omissions (t-score)	58.42±16.35	57.01±15.67	57.35±17.76	F_2,200_ = 0.36, p = 0.69	0.08	0.06
Commissions (t-score)	54.33±8.24	55.24±7.02	53.65±8.45	F_2,200_ = 0.59, p = 0.55	−0.11	0.08
Hit response time (t-score)	53.93±12.24	51.34±11.08	53.88±11.77	F_2,200_ = 1.57, p = 0.21	0.22	0.004
Hit response time standard error	59.61±10.83	56.99±10.51	58.20±10.52	F_2,200_ = 1.97, p = 0.14	0.24	0.13
Variability of standard errors	58.24±9.64	56.31±9.10	57.11±10.08	F_2,200_ = 1.12, p = 0.32	0.24	0.11
Response style (t-score)	53.36±9.64	52.61±9.74	51.62±7.55	F_2,200_ = 0.60, p = 0.54	0.07	0.20
Perseveration	76.81±39.39	76.04±36.48	68.71±36.01	F_2,200_ = 0.74, p = 0.47	0.02	0.21
Hit RT block change	52.90±11.84	51.86±13.12	49.98±13.83	F_2,200_ = 0.84, p = 0.43	0.08	0.22
Hit SE block change	52.72±9.59	52.21±10.42	49.14±11.84	F_2,200_ = 1.59, p = 0.20	0.05	0.33
Overall index	7.24±10.08	4.70±7.03	6.27±9.86	F_2,200_ = 1.92, p = 0.14	0.29	0.09

* *Values are mean ± SD unless otherwise specified. WISC-III = Wechsler Intelligence Scale for Children 3rd edition scores. S scores = standardized Scores.*

**Table 3:** Motivational style and Motor traits of ADHD children stratified according to BMI categories.

	Normal-weight n = 168	Over-weight n = 57	Obese n = 59	Test statistic and p-value	Effect size (Cohen's *d*)	
					Normal- weight vs. Over-weight	Normal- weight vs.Obese
**Motivational style** **evaluation**						
***Choice Delay Task***						
Total score	28.98±5.20	28.43±5.61	28.98±5.78	F_2,201_ = 0.16, p = 0.84	0.10	0.00
***Task-engagement Traits***						
RASS total score	55.22±25.50	54.05±26.79	50.83±28.45	F_2,199_ = 0.22, p = 0.80	0.04	−0.57
RASS vocalization score	6.55±7.48	6.33±7.07	6.24±8.41	F_2,199_ = 0.32, p = 0.96	0.03	0.03
RASS fidgeting score	12.84±7.09	13.61±8.47	14.32±7.22	F_2,199_ = 1.35, p = 0.26	−0.09	−0.20
RASS off task score	15.02±8.76	13.87±9.83	13.37±8.58	F_2,199_ = 0.46, p = 0.63	0.12	0.19
RASS plays with object score	14.75±8.55	14.47±9.01	12.97±8.67	F_2,199_ = 0.40, p = 0.67	0.03	0.20
RASS out of seat score	5.97±5.95	5.75±6.44	3.97±5.30	F_2,199_ = 1.99, p = 0.13	0.03	0.35
**Motor activity evaluation**						
***Actigraphy testing***						
Motor activity (Av. score)	85.23±42.68	97.67±47.95	81.76±58.41	F_2,190_ = 0.96, p = 0.38	−0.27	0.06
Motor activity (SD. score)	93.33±39.65	100.60±40.67	86.57±43.51	F_2,190_ = 0.85, p = 0.42	−0.18	0.16

* *Values are mean ± SD unless otherwise specified. Scores = standardized Scores. RASS  = Restricted Academic Situation Scale.*

*Av. Scores  =  average score, SD. Scores  =  standard deviation score.*

## Discussion

It has been reported that children with ADHD have elevated risk for obesity in both epidemiological [38] and clinical samples [37], [70], [71]. Given the well established health risks associated with childhood obesity [72], [73], it is important to understand the socio-demographic, neuropsychological and emotional determinants of the relation between obesity and ADHD.

Different theoretical models have proposed that ADHD and body mass regulation disorders share common pathophysiological underpinnings. From a neuropsychological point of view, it is suggested that ADHD and obesity stem from impairments in individual's self-regulation [11], [12]. More specifically, deficits in Executive Functions [13], [74], dysfunctional motivational regulation systems [75] and aberrant goal directed motor activity regulated by brain dopamine systems are believed to be implicated in both disorders. Supporting this line of argument, neuroimaging data in ADHD and obese subjects report commonality in brain structural abnormalities, including in the frontal cortex [33], [34], [35], [36], a locus considered to be important for self-regulation and EFs. In addition, ADHD subjects have been shown to have reduced DA receptor binding capacity in the hypothalamus, which controls for satiety and hunger [76].

The primary objective of the present study was to investigate the relation between body weight and cognitive, emotional, and motor activity characteristics in a large sample of children with ADHD. Results from our study do not identify statistically significant differences between weight groups (normal, over, and obese categories) in children with ADHD with respect to neurocognitive measures tapping in executive functions, motivational style indices, and overall motor activity. These findings contrast with the results of the only other study examining the association between EF and weight in children with ADHD [12]. More specifically, Graziano et al (2011) showed that children who performed poorly on a neuropsychological battery had greater BMI *z*-scores (overweight and obese) compared with children who performed better on the neuropsychological battery. Several factors may explain this discrepancy between the two studies. Compared to Graziano and colleagues, the current study has a much larger sample size (n = 80 versus n = 284), the participants have a narrower age range (4.5–18 years versus 6–12 years), and the neurocognitive assessment is more comprehensive in the present study. Unlike Graziano and colleagues, our participants were not taking any psychostimulant medication for at least one week prior to the neurocognitive assessments. Further, our statistical analysis model used parental (both mothers and fathers) age at child birth, family income status, MSDP, and prior history of treatment with psychostimulants as covariates to prevent potential confounding effects on the result outcomes, which was not the case in Graziano et al. study. In addition, the use of different neuropsychological tests indexing various EF domains in children with ADHD could also contribute to incongruent results. Contrary to Graziano et al. study where all cognitive domains were reduce into one single factor, in the present study; we elected to explore separately all performances assessed by various tests in order to identify any specific differences between the three groups. These analyses did not identify any significant differences, including in neuropsychological tests common to our study and Graziano and colleagues' work (Color-Word Interference Test).

The present study identified a strong association between socioeconomic (SE) characteristics and overweight and obesity status in children with ADHD, including parental age at child birth, MSDP and annual family income. These results are in line with results of previous reports in children from the general population exploring the role of socioeconomic, geographic and environmental factors in influencing body weight gain and fat distribution [43]. In developed countries, low SES strongly predicts obesity [77], [78] and the largest increase in obesity is observed in individuals living within the defined range of poverty [79]. Further, several lifestyle factors associated with obesity have a strong impact on children and adolescents belonging to low SES [80], [81], [82]. For instance, younger children having limited accesses to healthy foods, recreational venues and safe housing are 20–60% more likely to be obese/overweight compared to others [83], [84], [85], [86]. Due to increased financial burden, low SES families may not be able to afford to pay for their children's involvement in any formal sport/recreation activities, and these children may have limited access to safer parks or recreational facilities because they reside in poor neighborhoods [87]. This activity limitation prevents low SES child's engagement in a healthy, active lifestyle, and thus increases the chances of being overweight relative to their affluent high SES peers. Additionally, a diet with fruits and vegetables is highly recommended as part of a healthy lifestyle for growing children, but this may be less affordable for low SES families, resulting in poor nutrition and unhealthy weight gain. Consequently, it is plausible that lower SES is the main risk factor promoting overweight/obesity in children with ADHD.

Like ADHD [88], [89], weight gain/obesity is a multifaceted phenotype depending on complex interactions between genetic and environmental factors [5], [90]. More research in larger independent samples is recommended to further explore the complex relations between gene-environment-obesity amongst children with ADHD.

Our study has a number of strengths. First, to our knowledge, this is the largest and most comprehensive study examining the relations between weight status/BMI and neurocognitive profiles, motivational status and motor activity in children with ADHD. In addition, all clinical, neurocognitive, motivational and motor activity assessments were carried out while the children were not taking any medication (1 week wash out period).

The main limitations of this study also need to be considered. This study could not investigate the moderating effects of physical activity patterns [91] and eating habits/preferences [92] on the relation between body weight and ADHD due to unavailability of data. Future studies should address this issue, given the link between physical activity, eating preferences, obesity and ADHD. Further, our research design investigated BMI which is a generalized measure of body mass and lacked more direct and objective measures of obesity for example, underwater weighing, skin folds, etc. Finally, a link between altered sleep patterns, obesity and ADHD has been recently suggested [93], [94], given the unavailability of the sleep data in this sample, we could not investigate this potential interaction.

To summarize, our results show that childhood obesity in ADHD is associated with specific socioeconomic characteristics but not associated with impairments in self-regulation characteristics. These results do not support previous theories suggesting that impaired self-regulation promotes obesity in ADHD. Consequently, changing unhealthy life style amongst low SES children should receive more attention in future research, particularly those aiming at preventing childhood obesity amongst ADHD with children.
